# Emergence and repeatability of leadership and coordinated motion in fish shoals

**DOI:** 10.1093/beheco/arab108

**Published:** 2021-09-25

**Authors:** Dimitra G Georgopoulou, Andrew J King, Rowan M Brown, Ines Fürtbauer

**Affiliations:** 1 College of Engineering, Swansea University, SA1 8EN Swansea, UK; 2 Department of Biosciences, College of Science, Swansea University, SA2 8PP Swansea, UK

**Keywords:** collective behavior, coordination, emergence, leadership, phase transition, shoaling, time-depth

## Abstract

Studies of self-organizing groups like schools of fish or flocks of birds have sought to uncover the behavioral rules individuals use (local-level interactions) to coordinate their motion (global-level patterns). However, empirical studies tend to focus on short-term or one-off observations where coordination has already been established or describe transitions between different coordinated states. As a result, we have a poor understanding of how behavioral rules develop and are maintained in groups. Here, we study the emergence and repeatability of coordinated motion in shoals of stickleback fish (*Gasterosteus aculeatus*). Shoals were introduced to a simple environment, where their spatio-temporal position was deduced via video analysis. Using directional correlation between fish velocities and wavelet analysis of fish positions, we demonstrate how shoals that are initially uncoordinated in their motion quickly transition to a coordinated state with defined individual leader-follower roles. The identities of leaders and followers were repeatable across two trials, and coordination was reached more quickly during the second trial and by groups of fish with higher activity levels (tested before trials). The rapid emergence of coordinated motion and repeatability of social roles in stickleback fish shoals may act to reduce uncertainty of social interactions in the wild, where individuals live in a system with high fission-fusion dynamics and non-random patterns of association.

## Teaser

The coordinated motion of schools of fish and flocks of birds is fascinating to watch. Scientists have uncovered the interactions that produce these stunning patterns of motion, but focus on situations where coordination has already been established. Here we show that stickleback fish achieve coordinated motion by quickly establishing defined roles where some fish act as leaders and others as followers, and these roles are the same when the same fish are tested again.

## INTRODUCTION

Studies of self-organizing groups like schools of fish or flocks of birds have sought to uncover the behavioral rules individuals use (local-level interactions) to coordinate their motion (global-level patterns) (Couzin et al. 2002; Herbert-Read 2016). For example, experimental work and theoretical models have shown that individuals monitor and respond to nearby neighbors resulting in the emergence of coordinated motion in groups of insects (e.g., Kelley and Ouellette 2013; Attanasi et al. 2014), fish (e.g., Herbert-Read et al. 2011; Katz et al. 2011), birds (e.g., Cavagna et al. 2010; Bialek et al. 2012; Pettit et al. 2013; Ling et al. 2019), and ungulates (e.g. King et al. 2012; Torney et al. 2018). However, studies tend to examine “snap-shots” of collective behavior where coordination has already been established, or describe transitions between different coordinated states (Buhl et al. 2006; Tunstrøm et al. 2013). In doing so, only few works to date study the emergence or repeatability of coordination in biological systems (Buhl et al. 2006; Dyson et al. 2015; Murakami et al. 2017).

Individuals that live in groups often start (or re-start) interacting with one another from random or disorganized positions (see Biro et al. 2016 for a review). For example, a sudden predator attack upon a group of prey can spread individuals to new locations or environments, resulting in a completely different pattern of association (Herbert-Read et al. 2017). Interaction networks can also be interrupted when individuals have conflicting information (Merkle et al. 2015), and in social systems that exhibit high fission-fusion dynamics, individuals joining and leaving groups creates uncertainty in their social environment (Kelley et al. 2011; Ramos-Fernandez et al. 2018). In each of these contexts, coordination of individuals’ behavior in space and over time is disrupted or halted completely. To understand how coordination is achieved in such contexts, it requires repeated observations of animal groups when groups are formed, or during periods of disorder, that occur prior to the onset of coordination (Biro et al. 2016).

Here, we study the emergence and repeatability of coordinated motion in three-spined stickleback fish (*Gasterosteus aculeatus*). Three-spined sticklebacks are small gregarious fish that have become key models for our understanding of collective animal behavior (e.g., Harcourt et al. 2009; Jolles et al. 2018; Ward et al. 2018; Fürtbauer et al. 2020). Previous work has shown three-spined sticklebacks have defined leader-follower roles enabling coordinated motion among individuals (e.g. Harcourt et al. 2009; Nakayama et al. 2012, 2016; Hansen et al. 2016; Bevan et al. 2018; Jolles et al. 2020). We, therefore, expected leader-follower dynamics within shoals (prediction 1) affording coordinated motion (prediction 2). To identify leader-follower roles and coordinated motion, we tracked the motion of fish via video analysis and used the correlation among fish’s velocities through time (Nagy et al. 2010; Strandburg-Peshkin et al 2018; Fürtbauer et al. 2020) in combination with Wavelet analysis (Daubechies 1990; Torrence and Compo 1995; Gaucherel 2011; Aguiar-Conraria and Soares 2014). We did not anticipate fish leader-follower roles and group coordination to be instantaneous, but instead expected to see a transition from a disordered (uncoordinated, non-shoaling) to an ordered (coordinated, shoaling) state (prediction 3) since fish would need to (re-) establish social interactions/roles (Kelley et al. 2011; Borner et al. 2015; Merkle et al. 2015; Nadler et al. 2016). We, therefore, focused our analyses at the start of trials when fish shoals were introduced to a simple environment.

We also expected any leader-follower roles identified to be repeatable (prediction 4), since in a variety of shoaling fish species individuals show consistency in their tendency to act as leaders and followers (e.g., guppies, *Poecilia reticulate:*Ioannou et al. 2017; mosquitofish, *Gambusia holbrooki*: Burns et al. 2012) and three-spined stickleback fish show repeatable individual differences in behavior (Bell 2005; Dingemanse et al. 2007; King et al. 2013) that modulate leadership in shoals (Bevan et al. 2018). We, therefore, observed groups of fish across two trials, allowing us to investigate the influence of specific individuals on shoal motion over time.

Finally, leadership and followership roles can be related to a particular phenotype (Johnstone and Manica 2011; Jolles et al. 2020), and more active/exploratory three-spined stickleback fish are seen to adopt leader roles, whereas less active/exploratory fish adopt follower roles (Harcourt et al. 2009; Nakayama et al. 2012, 2016). We, therefore, tested whether inter-individual variation in fish motion when in a “start-box” before trials predicted fish leadership during trials (prediction 5). Furthermore, if leader-follower roles do exist and are linked to group coordination (see above) then we expected that coordination would be achieved quicker in the second trial (prediction 6) indicating a learning effect as the fish habituate to their environment and each other (Biro et al. 2016).

## METHODS

### Study subjects

Three-spined stickleback fish, wild‐caught from a pond on Swansea University Campus were initially housed in a large holding tank (30 × 39 × 122 cm), containing gravel substrate, plants, and driftwood. Fish were kept at a constant temperature/photoperiod regime (16°C/8:16 h light:dark) in which they remain reproductively quiescent (e.g., Katsiadaki et al. 2006; King et al. 2013). One week prior to behavioral tests, *n* = 30 fish (body mass: mean ± SD = 1.09 ± 0.16 g) were randomly chosen and transferred to individual 2.8 L gravel‐lined aerated tanks in which they were housed throughout the entire test period. Fish were fed once daily between 08.30 AM and 09.00 AM with defrosted bloodworms. All procedures described were approved by Swansea University’s Ethics Committee (IP‐1213‐3), and data on fish motion described in this paper have previously been used in combination with physiological measures to investigate androgen responsiveness and shoaling dynamics (Fürtbauer et al. 2020).

### Behavioral observations

**Figure 1:**
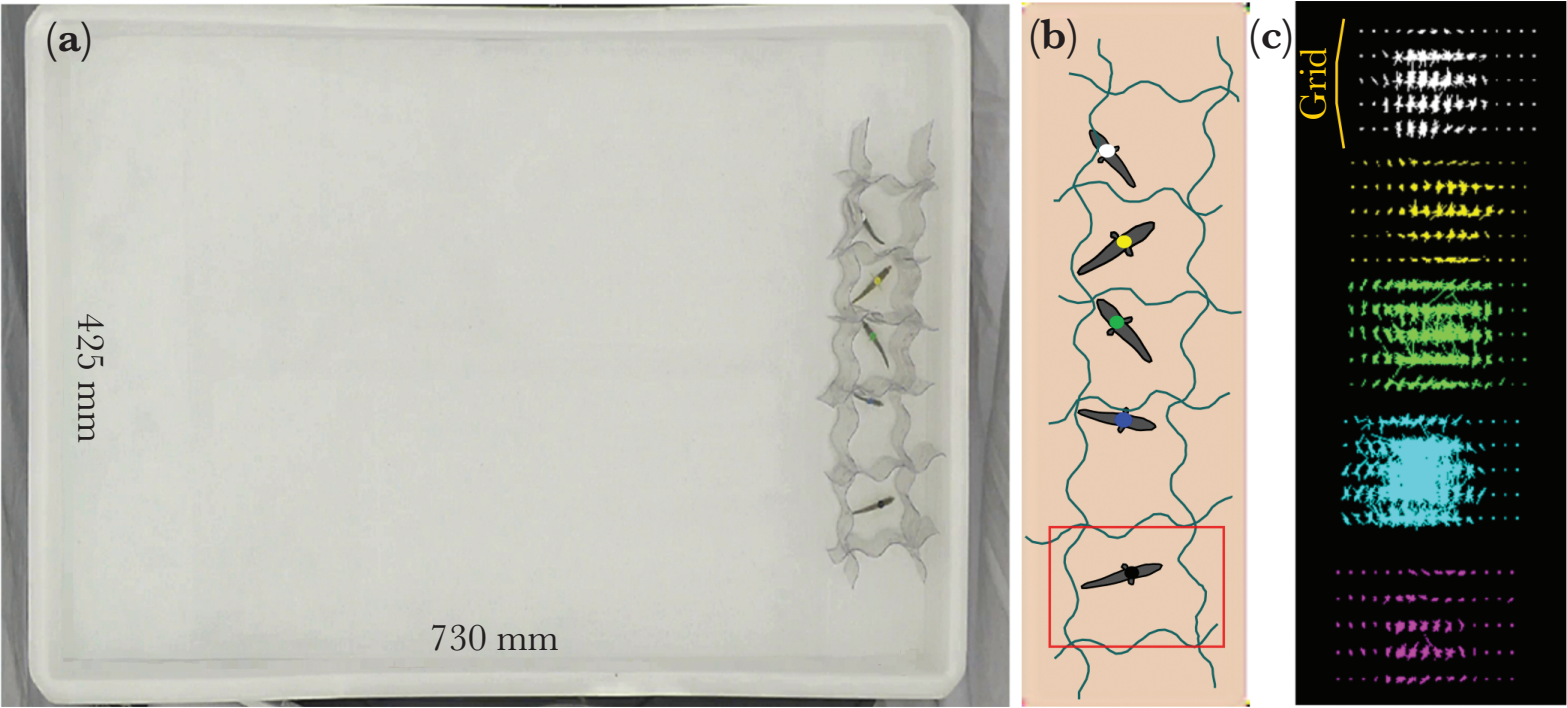
(a) The test arena (730 mm x 425 mm). Pictured is a group of fish in their start boxes (7.5 cm x 7.5 cm box for each fish); fish stayed in the start boxes for 5 min prior to being released into the full test arena. (b) A schematic representation of the transparent start boxes, fish and their colored identity tags. The red rectangle provides an example of the area used to determine fish motion in that box in the 5 min before free-swimming. (c) A schematic representation of the grid points that were used to detect fish motion in the start boxes. In this example the fish with the blue tag was identified as most active.

Singly housed fish were tagged for identification using spine-mounted colored plastic disc-shaped tags (Yellow, Green, Blue, Black, and White; Figure 1) (Webster and Laland 2009; Hansen et al. 2016; Fürtbauer et al. 2020) and randomly assigned to six shoals of *n* = 5 individuals. Several hours after attaching the tags the groups were filmed free-swimming in a rectangular test arena (42.5 × 73 cm) lined with white silica and filled with water to a depth of 5cm to constrain fish movement to mostly in two dimensions (Figure 1a). Fish were initially placed inside semi-transparent individual “start boxes” inside the test arena for 5 min (Figure 1b). The boxes were then removed, and fish swam freely for 20 min. The test arena was surrounded by a custom-built aluminum frame and white screen (PhotoSEL BK13CW White Screen) and a Panasonic HDC-SD60 HD video camera (Panasonic Corporation of North America, Secaucus, NJ) positioned above the test arena filmed the fish at a rate of 50 frames per second (fps). Four photographer’s lights (each with 4 × 25W, 240V, 6400K True Day light bulbs) lit the test arena from the outside dispersing light evenly over the arenas and enabling best conditions for video recording. The process described above was repeated, 24 h later, with the same group compositions. Although spine-mounted tags are shown not to affect fish activity or shoaling behavior (Webster and Laland 2009), because tagging is quick and reversible we chose to remove tags from fish after trial one, and re-attach them before trial two, ensuring that the protocol before each trial was identical.

### Tracking fish

Recent work with our study population of stickleback fish has shown that fine-scale measures of fish motion and broad-scale behavioral parameters that are commonly used in animal personality research are broadly equivalent (Bailey et al. 2021). We, therefore, measured fish motion in their start boxes (Figure 1c) using a custom-made routine (implemented in C++ using OPENCV library, Bradski 2000) and expected this to represent a measure of fish “activity”. First, the boundary of the start box was defined, and this area was overlaid with a uniform grid. Second, optical flow (Lucas and Kanade 1981) was calculated to detect motion at each of the grid points, and the sum of these motion events within each start box was used to provide a measure of individual fish motion (full details are provided in Supplementary Material). We also produced a standardized measure of motion, calculated as the measured fish motion divided by the sum of the levels of motion recorded for all fish within a group (referred to as “normalized box motion”).

To quantify the trajectories of each fish during free-swimming, we converted the pixel coordinates using the edges of the arena as reference points and developed a tracking algorithm that allowed segmentation of each fish (using OPENCV library in C++). This method was based on the color differences of fish tags and produced positional data using a Gaussian filter over 10 time-frames (further details are provided in Fürtbauer et al. (2020) and the Supplementary Material).

### Leadership

To test for leader-follower dynamics within shoals (prediction 1), we first calculated the correlation in velocity (*C*_*ij*_(*τ*)) between fish dyads *i* and *j* (e.g. Nagy et al. 2013; King et al. 2015; Strandburg-Peshkin et al. 2018; Fürtbauer et al. 2020) as follows (equation 1):


$$\begin{array}{l} C_{ij}\left(\tau \right)={〈{\vec{v}}_{i}\left(t\right)\cdot {\vec{v}}_{j}\left(t+\tau \right)〉}_{t_{w}} \end{array}$$ (1)

Where ${\vec{v}}_{i}\left(t\right)$ is the normalized velocity of fish *i* at time *t*, and ${\vec{v}}_{j}\left(t+\tau \right)$ is the normalized velocity of fish *j* measured relatively at a time lag, *τ*. The dot product calculates the directional correlation, where the limits, 0 and 1, correspond to no correlation and perfect correlation of fish *i* to fish *j* at time, *t* + *τ* respectively averaged over the time interval *t*_*W*_. We calculated *C*_*ij*_, for all dyads and recorded the maximum value of the directional correlation (CV*) as follows (equation 2):


$$\begin{array}{l} CV^{*}\left(t\right)=\max\left|C_{ij}\left(\tau \right)\right|\tau \in \left[-5\;\;5\right]\forall i,\;\;j,i\neq j \end{array}$$ (2)

Where CV* is the maximum directional correlation of fish *i* with fish *j* at time delay *τ*. If CV* occurrs at positive delay time *τ* > 0, this indicates that fish i’s direction is copied by fish j and, thus, the focal fish i leads fish j. Otherwise, if CV* occurs at *τ* < 0, the focal fish i copies j’ s direction, and follows fish j. All occasions that fish i’s direction was copied by fish j were recorded as leadership events. The directional correlations (and consequently leadership events) were calculated for *N* = *total duration (secs)*/4 secs non-overlaping intervals, to avoid overestimation of leadership. The total number of leadership events identified was divided by *N* to provide the frequency of leadership for each fish within a trial. No spatial filter was applied when identifying leadership events since fish were seen to respond to each other’s movements across the whole area (Supplementary Movies S1–S5).

### Coordination

We expected leader-follower dynamics (above, prediction 1) to afford group coordination (prediction 2) but did not expect this to be instantaneous (prediction 3). We, therefore, searched for evidence of a transition from a disordered to an ordered state, as measured by the change in mean CV*. To do this we used the “changepoint” package in R 3.4.4 (Killick and Eckley 2014) to detect the first significant change (here, an increase) in mean CV* as a function of time, which we took to indicate fish movements had reached a coordinated state (i.e. shoaling). Change points were calculated using different averaging time windows (*tw*) and signal lengths for calculating mean CV* and these were found to be stable when using different averaging time windows (Supplementary Figures S2 and S3); we present results using *t*_*w*_ = 55 s (Equation 2) and a signal length of 120 s for most trials (see Supplementary Material for details). We also used wavelet analysis to characterize fish oscillatory movements (shoaling) around the tank. Using Matlab function “cwt” we transformed the *x*(*t*) and *y*(*t*) coordinates of the fish into frequencies and expected significant changes in mean CV* to coincide with an emergence of a dominant frequency corresponding to the time interval needed for the shoal to travel around the tank.

### Statistical analysis

To test predictions 4–6, we used linear mixed models (LMMs) implemented in R (lmertest, R Core Team, 2013), and permutation tests implemented in Matlab (2017). Models’ diagnostics were checked and, where required, variables transformed with an appropriate Box-Cox power transformation to normalize model residuals.

To test if leader-follower roles are consistent for each fish observed across trials (prediction 4) we fitted leadership scores observed in trial two as our response variable and fitted the leadership scores observed in trial one as a fixed effect. We ran models for our time periods identified as disordered state (LMM1) and ordered state (LMM2). Group identity was fitted as a random effect in both models. We expected a significant positive correlation between frequencies of leadership only after the onset of coordination (LMM2) but not before (LMM1).

To test if inter-individual variation in fish motion prior to free-swimming predicted the frequency of leadership (prediction 5) we first confirmed that box motion scores were (i) independent of neighbor motion and fish position in the start box array (Figure 1), and (ii) repeatable. To test (i) we used permutation analyses (Van Rossum and Drake Jr 1995) to test for significant differences in fish motion between neighbors and non-neighbors within each group, and across the entire dataset. In both cases, we calculated the difference in the motion of neighbors and non-neighbors (within groups or across the dataset), calculated a test statistic, and constructed a distribution using 20000 resamples without replacement (see Supplementary Material for more details). To test (ii) we fitted a model (LMM3) with motion in start box (trial two) as our response variable, motion in start box (trial one) as the fixed effect, and group identity as a random effect. We tested fish motion scores and standardized motion scores, and results were equivalent, with fish motion in the start box was repeatable across trials (Supplementary Figure S4a) and independent of neighbor motion (Supplementary Figure S4b). We, therefore, proceeded to run a model (LMM4) with frequency of leadership as our response variable, fish motion in the start box (continuous), and trial (one, two) as fixed effects, and with group identity as a random effect.

To test for a learning effect (prediction 6) whereby coordination occurs quicker in the second trial, we ran a final model (LMM5) with change point time as the response variable, trial (one, two) as a fixed effect, and group as a random effect. We also considered the possibility that the overall level of motion shown by the (group of) fish may influence the onset of coordination on the basis that more active fish may become coordinated more quickly, and so also fitted mean fish motion in the start box as an additional fixed effect. We ran LMM5 twice, including and excluding the data from Group B because they took much longer to coordinate in trial 2 compared to other groups for an unknown reason (Supplementary Figure S3).

## RESULTS

### Leader-follower dynamics

**Figure 2:**
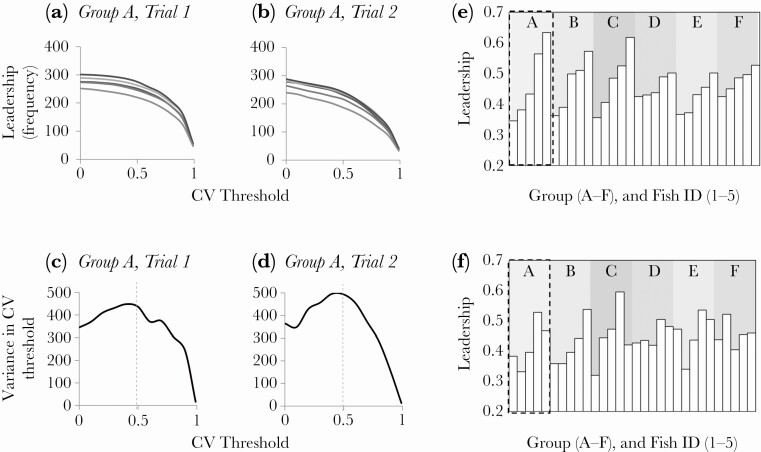
Examples of the number of leadership events for fish in Group A (each fish is represented by a different shade) during Trial 1 (a) and Trial 2 (b), as a function of different CV* thresholds and where CV* = 1 indicates perfect correlation in the directions of fish i and fish j delayed in time τ; (c) and (d) show the variance in frequency of leadership between group individuals is maximized at values CV* > 0.5 for the data shown in (a) and (b); (e) and (f) show leadership within and across trials. Here, the number of occasions each fish led was divided by the sum of all leadership events within a group to provide as normalized leadership score for each fish. Fish ID is presented according to the leadership score in trial 1 and the order is maintained independent of leadership score in trial 2 for comparisons. Group A is highlighted by dashed lines and associated leadership data are given in Figures (a)-(d).

Leadership scores were variable within shoals (Figure 2a, b), and this variability was maximized when a correlation between two fishes' velocities (CV*) was greater than 0.5 (Figure 2c, d). In each group, fish could be ranked 1–5 according to the number of times they led group-mates (Figure 2e, f), thus providing evidence for leader-follower dynamics (prediction 1).

### Transition to coordination

**Figure 3:**
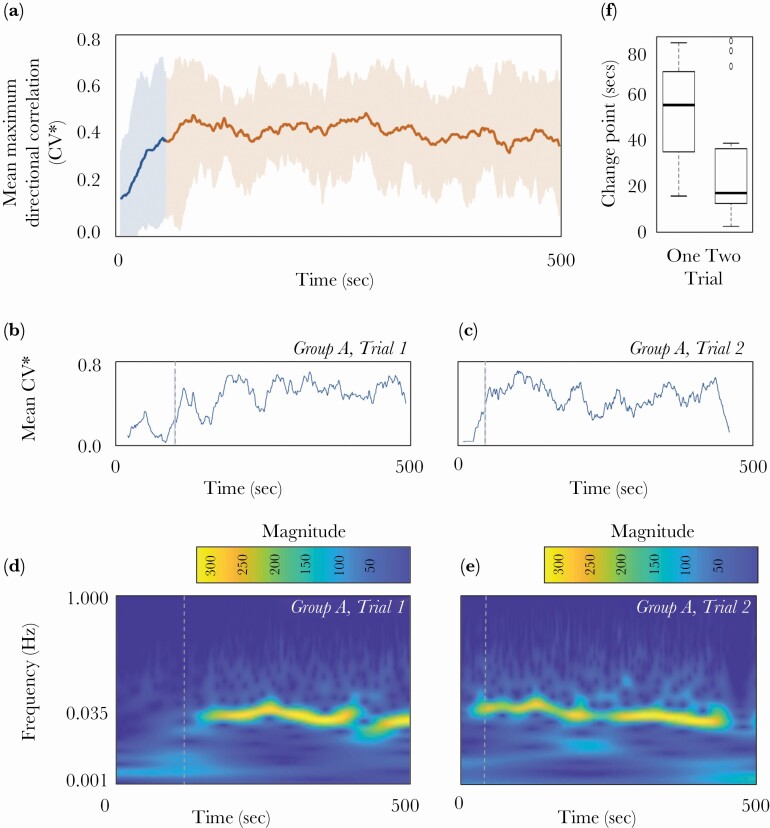
(a) Emergence of coordination identified by mean maximum directional correlation (CV*, line) and min/max CV* range (shaded area) across all studied fish groups over time. Mean CV* change point time = 40.6 secs and is indicated by a change in color from blue to red); (b) and (c) show mean CV* over time for Group A trial 1 and trial 2 and identified change points (dashed vertical line). The same plot for all groups and trials are given in Supplementary Figure S5; (d) and (e) provide continuous wavelet transform scalograms for Group A in trial 1 and trial 2. The different colors represent the correlation between the scaled/translated Morlet wavelets and the mean position of the group (expressed as the mean distance of the fish from a fixed tank point) in time. Warmer colors (yellow) indicate higher correlation. The yellow pattern shows the dominant frequency of this oscillatory motion which is around 0.035 Hz and indicates the emergence of oscillatory movement around the tank with period of 25–30 s confirming video observations (Supplementary Movies S1–S5 provide examples). The same plots for all groups and trials are provided in Supplementary Figure S6. (f) Box plot showing mean (vertical lines) quartiles (box) and 95% range (whiskers) for detected change points in during trial 1 and trial 2. Change point for Group B trial 2 was >300 s and not shown.

Leader-follower dynamics afforded coordinated motion (prediction 2), but this was not instantaneous. Taking CV* to indicate level of coordination within the shoal, we were able to identify a transition from a disordered to an ordered state within 1.5 min (upper time limit) of fish being released from their start boxes (mean CV* change point time = 40.63 s, range 10.58–88.1 s; Figure 3a) supporting prediction 3. Once coordinated, fish tended to move together around the edges of the test arena (Supplementary Movies S1–S5 provide examples), and this oscillatory motion resulted in a frequency maximized at 0.035*Hz* (Figure 3b, c), which is the reciprocal of the length scale of the fish tank and matched the timing of identified change points in CV* (Figure 3d, e).

### Repeatability of leadership

**Figure 4:**
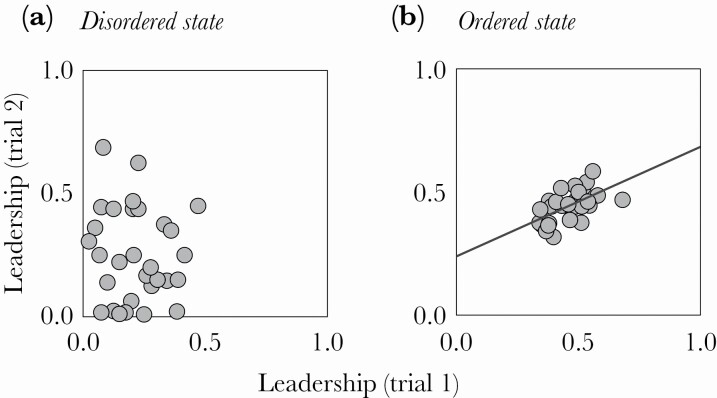
Relationship between individual fish leadership scores in trial 1 and trial 2, for all groups, during (a) disordered, non-shoaling states, and (b) ordered, shoaling states. The line in (b) represents the effect predicted by LMM2 (see methods). Figure 3 provides further information on defining the two states.

Prior to groups achieving coordination (i.e., in a disordered state), leadership (occasions fish *i* led fish *j*) was not correlated across trials (Figure 4a; LMM1: Effect ± SE = –0.16 ± 0.26, *t*-value = –0.62, *P* = 0.54). However, when coordination was achieved (i.e., during the ordered state) fish leadership was highly correlated across the two trials (Figure 4b; LMM2: Effect ± SE = 0.42 ± 0.11, *t-*value = 3.85, *P* <0.01), in support of prediction 4. The motion of fish in the start box prior to free swimming were repeatable across trials (Supplementary Figure S4a; LMM3: Effect ± SE = 0.32 ± 0.13, *t-*value = 2.41, *P* = 0.02), were unrelated to box position (Supplementary Figure S4b; Supplementary Table S2) and did not predict individual leadership (LMM4: Effect ± SE = –0.001 ± 0.006, *t*-value = –0.021, *P* = 0.83), contrary to prediction 5.

### Time to coordination

Groups achieved coordination faster in trial two than in trial one (Figure 3f; LMM5: Effect ± SE = -0.28 ±0.03, t-value = -8.07, P<0.01) in support of prediction 6, and we also found that groups of fish with higher mean motion in the start boxes were faster to coordinate (LMM5: Effect ± SE = -2.62± 1.89, *t-*value = –0.74, *P* = 0.009).

## DISCUSSION

We show that shoals of stickleback fish introduced to a simple environment are initially uncoordinated in their motion, but quickly transition to a coordinated state with defined individual leader-follower roles. The identities of leaders and followers were repeatable across two trials, and coordination was reached more quickly during the second trial, and by groups of fish with higher mean levels of motion recorded before free-swimming trials commenced.

Defined leader-follower roles were repeatable across two observations. The adoption of specific leader-follower roles within stickleback fish shoals is in keeping with previous work (e.g. Hansen et al. 2016; Bevan et al. 2018; Jolles et al. 2020). Indeed, experiments with pairs of stickleback fish (Harcourt et al. 2009; Nakayama et al. 2012, 2016) have shown that individuals that are more likely to leave cover and explore their environment when tested alone (“bolder” individuals) are more likely to lead their partners, whereas fish that are less likely to leave cover when alone (“shyer” individuals) follow their partners motion and elicit greater leadership tendencies in their bold partners. Other work has shown the likelihood of individuals to approach conspecifics is negatively correlated with an individual’s tendency to leading stickleback dyads and shoals (Jolles et al. 2014, 2017). In this study, we tested whether fish motion in small start boxes prior to the start of trials predicted leadership. Although we found repeatable individual differences in the level of motion observed, this did not predict frequency of leadership. The lack of a link between motion in the start box and leadership could indicate that motion captured prior to free-swimming does not reflect “activity” as measured in other behavioral studies (Carter et al. 2013). However, this finding is similar to other work with stickleback fish shoals (of the same shoal size) that did not find links between exploratory tendency and leadership (Jolles et al. 2017), and a recent study of our fish population has found fine-scale motion and broad-scale behavioral parameters are broadly equivalent, suggesting value in this approach (Bailey et al. 2021). Future work should now focus on between-individual variation in how fish balance goal-oriented movement and socially oriented behaviors (Conradt et al. 2009) and attempt to measure this trade-off (sticking with others versus moving away from them) in-situ during controlled experiments, and in different contexts (e.g. responses to unpredictable food resources: MacGregor et al. 2020).

We show a rapid emergence of coordinated motion in the stickleback fish groups. At the start of trials, the correlation in velocity among fish was low, no consistent leader-follower dynamics were observed, and wavelet analysis showed fish were slow-moving without a consistent oscillatory pattern. Then, relatively quickly the mean correlation in velocity among fish increased, fish moved together tending to cycle around the edges of the arena, and consistent leader-follower dynamics were present (see Supplementary Movies S1–S5 for examples). Combining directional correlation in fish velocities with wavelet analysis offers promise for future work. For example, wavelet analysis can be used to decompose a signal (e.g., positional data) into its frequency characteristics in a time localized manner thus providing information on the major types of collective behavior displayed by individuals or groups. Wavelet analysis may thus provide a way of characterizing a group’s collective state that is not determined by one aspect of behavior (e.g. group polarization: Tunstrøm et al. 2013). Examination of local dynamics (e.g., directional correlation in velocity) for different collective states as identified by wavelet analyses can then be used to test if local interaction rules are flexible or robust with respect to change (King et al. 2018). For example, in the laboratory, this could be changes in physical boundaries (Pinter-Wollman 2015), or in the wild moving from a closed habitat to an open habitat (King et al. 2009). If interaction rules are flexible, then individuals in animal collectives should adaptively change their behavior when faced with a change in environment. If interaction rules are robust, then individual behavior should persist (but perhaps be sub-optimal) when experiencing change. As yet, these sorts of questions are relatively unexplored, but are critical for understanding the impact of environmental features and changes in the environment upon the behaviors of individuals, groups, and species (Flood and Wong 2017; Snijders et al. 2017; King et al. 2018). Work investigating the role of the built environment in shaping collective outcomes in social insects offers ideas here, since methods and theory in this area are relatively well developed (e.g. Pinter-Wollman et al. 2017, 2018).

The onset of coordination was quicker during the second trial. It has been proposed that there should be a feedback loop between leadership, learning, and competence with the potential to affect improvements in collective performance over time (Biro et al. 2016). Our finding that coordination occurs more quickly in trial two indicates previously established leader-follower interactions might be reinforced at the start of the second trial. However, the fact that we also found that groups containing fish with higher levels of motion in their start boxes also achieved coordination faster, it may be that familiarity with the test arena during trial two resulted in overall quicker coordination in groups because fish began moving and interacting more quickly. To confirm the presence of such (collective) learning would require further repeat tests, involving changing environments so that fish are only learning about one another (and not their environment). For instance, a study of newly formed monk parakeet (*Myiopsitta monachus*) groups (*N* = 21 and 19) showed a feedback between behavior and knowledge (as inferred by model fitting and comparisons) that allowed groups to rapidly transition to large-scale order in aggressive interactions (Hobson and DeDeo 2015). This structuring happened in a manner that could not be accounted for by individual characteristics, or by the spatial position of individuals. Therefore, we suggest that further work on re-establishing directed interactions (e.g. leader-follower) as studied here, will allow us to determine if roles emerge as a consequence of differences in individual characteristics (e.g. size, speed) (Jolles et al. 2020), or by recognizing and monitoring the behaviors of those around them (King et al. 2011). The latter tends to be assumed for studies of collective behaviors in, for example, primates (King and Sueur 2011), whilst the former interpretation applies to studies of fish shoals or bird flocks (Killen et al. 2017). Our finding that coordination is reached more quickly in a second trial suggests a role for learning/memory (Biro et al. 2016) in the stickleback system – whether it be for their ecological and/or social environments.

In the simple and stable environment studied here, the dynamics on the collective behavior appear to stabilize quickly, but we do not know if the emergence of coordinated motion we see for our study fish is “fast” (though it intuitively seems to be). We, therefore, propose that the speed with which the fish achieve coordination may be useful in the wild where individuals form large groups exhibiting fission–fusion dynamics (Peuhkuri 1998; Couzin 2006) and may act to reduce uncertainty of social interactions (Sueur et al. 2011; Ramos-Fernandez et al. 2018). What now needs to be determined is whether the consistency of social roles (leader-follower dynamics) we see in our study is also repeatable when individuals find themselves in different social settings. For instance, if we create groups composed of all top-ranked and bottom-ranked leaders in our study groups, will individuals similarly order themselves with respect to leadership and quickly achieve coordination, or fail to effectively coordinate? Given that our small sample of randomly composed groups all showed similar local interaction rules and collective behaviors, it unlikely social roles are innate; instead they are likely to emerge through repeated interactions (King et al. 2018). Understanding how these roles emerge and change over time will be imperative to understanding individual- and group-level behavioral evolution (Bengston and Jandt 2014).

## Supplementary Material

arab108_suppl_Supplementary_Material

arab108_suppl_Supplementary_Movie_S1

arab108_suppl_Supplementary_Movie_S2

arab108_suppl_Supplementary_Movie_S3

arab108_suppl_Supplementary_Movie_S4

arab108_suppl_Supplementary_Movie_S5

## Notes

The authors thank AJK and IF research groups for discussion and Layla King for support. DGG thanks Danis Kiziridis for the fruitful discussions on the methodology.

Author contributions: I.F. designed the project and collected the data. D.G.G., M.R.B., A.J.K. and I.F. developed the methods for data analyses, and D.G.G. analysed data. D.G.G. wrote the first draft of the manuscript and all authors contributed to the writing of the final manuscript.

Data availability: Analyses reported in this article can be reproduced using the data provided by Georgopoulou et al. (2021).
