# Supplementary material for: Emergence and repeatability of leadership and coordinated motion in fish shoals
Source: Behav Ecol. 2021 Sep 25;33(1):47–54. doi: 10.1093/beheco/arab108 (PMC8857939; doi:10.1093/beheco/arab108)
Supplement: arab108_suppl_Supplementary_Material [file arab108_suppl_supplementary_material.docx]

Supplemental Material *for:*

**Emergence and repeatability of leadership and coordinated motion in fish shoals**

D. G. Georgopoulou, A. J. King, M. R. Brown, I. Fürtbauer

Contents

[SUPPLEMENTARY METHODS 2](#_Toc78806774)

[Code and analyses 2](#_Toc78806775)

[Detecting motion of fish in the starting boxes 2](#_Toc78806776)

[Permutation test 2](#_Toc78806777)

[Tracking fish trajectories during free-swimming 3](#_Toc78806778)

[Table S1. 3](#_Toc78806779)

[Figure S1. 4](#_Toc78806780)

[Change points in mean CV* 4](#_Toc78806781)

[Figure S2. 4](#_Toc78806782)

[Figure S3. 5](#_Toc78806783)

[SUPPLEMENTARY RESULTS 6](#_Toc78806784)

[Fish motion levels 6](#_Toc78806785)

[Table S2. 6](#_Toc78806786)

[Figure S4. 6](#_Toc78806787)

[Emergence of coordination (change points on CV*) 7](#_Toc78806788)

[Figure S5. 7](#_Toc78806789)

[Emergence of coordination (Wavelet analysis) 8](#_Toc78806790)

[Figure S6. 8](#_Toc78806791)

[References 9](#_Toc78806792)

# SUPPLEMENTARY METHODS

## Code and analyses

All code and analyses are available in Dryad repository: <https://doi.org/10.5061/dryad.nvx0k6drj>

## Detecting motion of fish in the starting boxes

The quantification of the box motion level was realized using a custom-made routine (in C++/OPENCV: Bradski, 2000) that includes the following steps: First, the outline of the region each fish is located is drawn and a 4x5 grid of points is automatically created that covers all the area of the box (Figure 1, main text). Second, the optical flow (Lucas and Kanade, 1981) algorithm is applied on the grid points to detect motion/activity at each of the points for the 5 minutes fish were in the start boxes. Optical flow methodology tracks specific features (points) in an image across multiple frames. It works on several assumptions: that the pixel intensities of an object do not change between consecutive frames and that the neighbouring pixels have similar motion. Every time a point motion is detected an activity event is detected. An activity index of fish $i$ and group $grp$ is defined as the sum of the activity events over the whole time interval T ( 0 <$T$< 5 mins):

$a_{grp}^{i}$ =$\sum_{t}^{T} \sum_{j}^{J} a_{t}^{j}$

where $a_{t}^{j}$= 1 when the j^th^ grid point at time t is activated and 0 otherwise. To compare motion of fish between groups, we also calculated each fish’s activity value divided by the sum of the activity levels of all fish of its group (normalized box activity, $\alpha_{grp}^{i}$), as:

$$\alpha_{norm}^{i}=\frac{\alpha_{grp}^{i}}{\frac{1}{N}\sum_{i}^{N} \alpha_{grp}^{i}}$$

where$\alpha_{grp}^{i}$ is the motion of fish $i$ and group *grp* and N is the total number of observations per group.

## Permutation test

We used permutation analysis to test if the motion of one fish could be affected by the motion of fish in neighboring boxes. The observed statistic was the subtraction between the motion difference of the neighbors and the motion difference of the non-neighbors, averaged over all individuals within the same group (method 1), or across all groups (method 2), as:

$$Statistic_{obs}= \left\langle\bar{{d_{n}}^{obs}}-\bar{{d_{nn}}^{obs}} \right\rangle_{grp}$$

where $\bar{d_{n}^{obs}}$ is the mean motion difference of the neighbours and $\bar{d}_{nn}^{obs}$ is the mean motion difference of the non-neighbors for a group of fish. Then analysis consisted of the following steps:

A raw motion value was randomly assigned to each individual of each group by resampling without replacement from all raw motion values. Then, the motion difference of neighbors and the motion difference of non- neighbors were calculated for all groups. The test statistic was defined as:

$$Statistic= \left\langle\bar{d_{n}}-\bar{d_{nn}} \right\rangle_{grp}$$

Then, the permutation distribution of the statistic was constructed using 20000 resamples without replacement and the observed statistic was compared against the permutation distribution and the p-value was calculated (using a two-tailed test).

## Tracking fish trajectories during free-swimming

To extract the trajectories of each fish, we developed a tracking routine based on OPENCV/ C++. Each fish was tagged with a specific colour. The segmentation of each fish was based on their colour differences. The colours used were black, yellow, green, blue and white. As the background was white and the illumination conditions were constant, segmenting the black, yellow, green and blue colours was straightforward. However, the white tag could not be segmented, but a different methodology was used to detect that fish. We worked on the HSV (hue, saturation, value) colour space (an alternative representation of the Red Blue Green colour space) to determine the range each colour tag has in the video. HSV colour space, like RGB, also consists of 3 matrices, the HUE, the SATURATION and the VALUE. The HUE represents the color, SATURATION represents the amount to which that respective colour is mixed with white and VALUE represents the amount to which that respective colour is mixed with black. In OPENCV, value ranges for HUE, SATURATION and VALUE are respectively 0 − 179, 0 − 255 and 0 − 255. We decided to use HSV colour space, because we know the colour values for each tag (i.e. Hue values). The Saturation and Value would change more because of changes in water due to the movement of fish or the exact position of the fish in the tank. As expected, the different colour tags were thresholded using non-overlapping distinct Hue range values for the different colours. The ranges are given in Table S1.

Table S1. Ranges of the HSV values used to segment the coloured discs. H stands for Hue values, i.e., the values that characterize the chroma/colour of each tag. S stands for saturation and V stands for Value. These two parameter values vary a lot (around 200 units).

| **Fish tags** | **H** | | **S** | | **V** | |
| --- | --- | --- | --- | --- | --- | --- |
|  | Min | Max | Min | Max | Min | Max |
| **Yellow** | 19 | 33 | 40 | 255 | 92 | 255 |
| **Green** | 43 | 74 | 50 | 255 | 72 | 255 |
| **Blue** | 76 | 179 | 43 | 252 | 30 | 162 |
| **Black** | 0 | 179 | 0 | 255 | 0 | 49 |
| **All fish** | 0 | 179 | 0 | 255 | 0 | 120 |

To segment the fish with the white tag we applied the following: we created a mask of all the five fish by segmenting the image using Otsu’s threshold (Figure S1a). We applied a bitwise and operation (a.k.a. an intersection) between the segmented image of each of the colour tags (Figure S1b) and the segmented image of the five fish to extract the mask of the four fish (Figure S1c). Finally, we used the last image as a mask to get only the objects that correspond to the white fish (Figure S1d). We grouped the remaining contours to get a solid object that corresponds to the white targeted fish.

Following the above procedure, an image with 5 segmented objects was obtained, where each object corresponds to one of the fish. The fish tracks are the collection of the positions (the centroid of the disk) of the segmented object that corresponds to a specific tag. In case of disk occlusions at a time frame $t$ the position of the fish is left empty, and these missing values were interpolated.

Figure S1. Image processing steps using OPENCV to extract the fish with white tag. (a) Initial segmentation using Otsu’s threshold. (b) The segmented coloured tags, after using simple threshold on the HSV space. (c) The result of the intersection of the two previous thresholded images. (d) Final result of the segmentation of the fish with the white tag.

## Change points in mean CV*

We expected significant changes in mean CV* value where groups began shoaling (ordered state). For this, we used the changepoint package in R (Kuznetsova, 2017), and, specifically the cpt.mean() function to detect a single changepoint in the $CV^{*}$ signal. We chose the CUSUM method, which has no distributional assumptions. As we were interested in detecting the initial change in coordination, we aimed at detecting the first only change in CV*. The detection of the single change point was affected by the inputted signal length, with longer signal lengths to detect change points that existed after the coordination event (Figure S2a). This is expected as the inputted signal was 10 minutes long and extra changes in coordination could happen during this time. In addition, the coefficient of variation of the mean/median of changepoints increased for increased signal lengths reaching a plateau (Figure S2b). From video observations we don’t expect initial onset of coordination to take place later than 2 mins. For this reason, we chose the signal length of 150 secs (where variation of the change point is also low) for the cases where coordination happened quickly. Finally, for the cases where coordination was established immediately after the release in the arena, the $CV^{*}$ values were higher at the start of trials. For these cases, when the signal had initial $CV^{*}> 0.3$ a shorter signal was used as an input to the function. This happened in trial 2 for fish dyads in groups D, E and F.

Figure S2. (a) The influence of the signal length on the detection of the changepoint (onset of coordination). The line indicates mean value of the change-points for different signal lengths. The shaded area is the coefficient of variation of the mean. (b) Coefficient of variation (percentage) of the mean (black) and median (green) of the change points. The coefficient of variation approaches a plateau for signal lengths over 100 secs.

We also tested how sensitive the change point detection was for the different time averaging windows, $t_{w}$, (where $t_{w}$ is used for the calculation of $C_{ij}$, and therefore the ${CV}^{*}$). Figure S3 shows that the change point is relatively stable for different time windows.

Figure S3. (a) and (b) show detection of the change-point of the ${CV}^{*}$ for different averaging time windows used to calculate the *CV* for the first and second set of trials. Solid lines indicate the mean change point detection while shaded lines indicate the standard error of the mean. Change point detection is robust to different time windows used when calculating$C_{ij}$.

# SUPPLEMENTARY RESULTS

## Fish motion levels

Table S2. Permutations testing for potential differences between observed motion levels among neighbouring fish and non-neighbouring fish for each group (Table S1a), and each trial (Table S1b). Shown are the test statistic, the observed statistic and the P-value. All tests are non-significant indicating fish position in the start box did not influence the detected fish motion.

Table S2a

| Trial | Group ID | Test Statistic | Observed | P-value |
| --- | --- | --- | --- | --- |
| Trial 1 | Group A | 1098.2 | 1179.8 | 0.63 |
|  | Group B | 630.8 | 453.5 | 0.28 |
|  | Group C | 260.8 | 2528.2 | 0.45 |
|  | Group D | 1431.8 | 1228.8 | 0.37 |
|  | Group E | 2361.4 | 1963 | 0.32 |
|  | Group F | 2156.6 | 2699.7 | 0.90 |
| Trial 2 | Group A | 3236.6 | 4384 | 0.93 |
|  | Group B | 2114.2 | 2523 | 0.76 |
|  | Group C | 1384.8 | 1199.5 | 0.33 |
|  | Group D | 1573.4 | 1351.3 | 0.37 |
|  | Group E | 1722.2 | 1361 | 0.18 |
|  | Group F | 1523.6 | 943.5 | 0.13 |

Table S2b

| Trial |  | Test Statistic | Observed | P-value |
| --- | --- | --- | --- | --- |
| 1 |  | 23.58 | -64.88 | 0.92 |
| 2 |  | 483.3 | 57.62 | 0.95 |
|  |  |  |  |  |

Figure S4. (a) Fish normalized motion in the start box “activity level” were repeatable across trials (Spearman’s rho = 0.399, p = 0.029, n = 30). Each dot indicates a fish, coloured by group ID (6 groups). (b) Boxplots illustrating similar differences in fish motion in the start box “box activity” between neighbo

rs and non-neighbors. The whiskers show the range of values, the boxes represent the interquartile range box, i.e. the middle 50% of the data.

## Emergence of coordination (change points on CV*)

Figure S5. The maximum directional correlation (CV*, black lines) for groups of fish over time with a vertical line (yellow) indicating the detected change point for the group, for trial one (a, c, e, g, i, k) and trial two (b, d, f, h, j, l).

## Emergence of coordination (Wavelet analysis)

Figure S6. Continuous wavelet transform scalograms (Matlab, 2017) which represent the percentage of energy for each coefficient of the different frequencies of our signal for fish positional data during free-swimming. Each row represents a group, with left column represents trial 1 (a, c, e, g, i, k), and right column represents data from trial 2 (b, d, f, h, j, l).

##

## References

Bradski G. 2000 The OpenCV Library. *Dr. Dobb’s J. Softw. Tools*

Kuznetsova A, Brockhoff PB, Christensen RHB. 2017lmerTest Package: Tests in Linear Mixed Effects Models.*Journal of Statistical Software,* 82(13), 1–26

Lucas BD, Kanade T. 1981 An Iterative Image Registration Technique with an Application to Stereo Vision. In *Proceedings of the 7th International Joint Conference on Artificial Intelligence - Volume 2*, pp. 674–679. San Francisco, CA, USA: Morgan Kaufmann Publishers Inc.

Matlab, 2017 MATLAB version 9.3.0.713579 (R2017b).

Van Rossum G, Drake Jr FL. 1995. Python reference manual. Centrum voor Wiskunde en Informatica Amsterdam.
